# Surface expression marker profile in colon cancer cell lines and sphere-derived cells suggests complexity in CD26^+^ cancer stem cells subsets

**DOI:** 10.1242/bio.041673

**Published:** 2019-07-08

**Authors:** Lorena Vázquez-Iglesias, Leticia Barcia-Castro, Marta Rodríguez-Quiroga, María Páez de la Cadena, Javier Rodríguez-Berrocal, Oscar J. Cordero

**Affiliations:** 1 Department of Biochemistry, Genetics and Immunology, Facultade de Bioloxía, Universidade de Vigo, 36200 Vigo, Galicia, Spain (EU); 2 Department of Biochemistry and Molecular Biology. CIBUS Building, Facultade de Bioloxía. Universidade de Santiago de Compostela, 15782 Santiago de Compostela, Galicia, Spain (EU)

**Keywords:** Colorectal cancer, Cancer stem cells, Epithelial-mesenchymal transition, Biomarkers, CD26, EpCAM, LGR5, E-cadherin, CD133

## Abstract

Taking advantage of eight established cell lines from colorectal cancer patients at different stages of the disease and the fact that all of them could form spheres, cell surface biomarkers of cancer stem cells and epithelial-mesenchymal transition were tested. The aim was to investigate cancer stem cells and metastatic stem cells in order to provide functional characterization of circulating tumor cells and promote the development of new anti-metastatic therapies. Our model showed an important heterogeneity in EpCAM, CD133, CD44, LGR5, CD26 and E-cadherin expression. We showed the presence of a subset of E-cadherin^+^ (some cells being E-cadherin^high^) expressing CD26^+^ (or CD26^high^) together with the well-known CSC markers LGR5 and EpCAM^high^, sometimes in the absence of CD44 or CD133. The already described CD26^+^/E-cadherin^low^ or ^negative^ and CD26^+^/EpCAM^−^/CD133^−^ subsets were also present. Cell division drastically affected the expression of all markers, in particular E-cadherin, so new-born cells resembled mesenchymal cells in surface staining. CD26 and/or dipeptidyl peptidase 4 inhibitors have already shown anti-metastatic effects in pre-clinical models, and the existence of these CD26^+^ subsets may help further research against cancer metastasis.

## Abstract (precis)

**Summary:** In our model of eight established cell lines from colorectal cancer patients we show the presence of different putative cancer stem cell (CSC) subsets with expression of CD26/DPP4.

## INTRODUCTION

Metastasis accounts for the vast majority of deaths due to cancer because even if the primary tumor has been perfectly removed by surgery, tumor cells can have disseminated and established themselves in distant locations (Oskarsson et al., 2014; Pantel et al., 2008; Liu et al., 2014a,b; Miranda-Lorenzo et al., 2014; Driessens et al., 2012). Cancer stem cells (CSCs) are the only tumor cell type with long-term self-renewal potential because of their microenvironmental niche (Oskarsson et al., 2014; Liu et al., 2014a,b; Miranda-Lorenzo et al., 2014), suggesting that metastatic stem cells (MetSCs) with tumor-initiating capacity already exist in the primary tumor (Oskarsson et al., 2014; de Sousa e Melo et al., 2017). Since metastasis does not rely on driver mutations, and genomic biomarkers are not useful for diagnosis (Vanharanta and Massagué, 2013), the possibility that MetSCs may be tracked is especially important for metastasis diagnosis and development of therapeutic approaches that kill them (Oskarsson et al., 2014).

Cell surface markers are powerful tools, for example, for isolating distinct cell populations from freshly harvested primary tumors (Miranda-Lorenzo et al., 2014) or blood. However, the expression levels of markers of CSCs change depending on environmental conditions; they are not reproducible across or even within similar tumor types, and they are not exclusive of a functional CSC phenotype (Miranda-Lorenzo et al., 2014; Driessens et al., 2012; de Sousa e Melo et al., 2017; Vanharanta and Massagué, 2013; Wicha et al., 2006). Thus, an important question is which markers should be used for CSC and MetSC characterization.

In colorectal cancer (CRC), MetSCs are already present in the primary tumor (de Sousa e Melo et al., 2017; Varela-Calviño and Cordero, 2015). Candidate markers for CRC MetSC characterization include CD166, CD29, CD24, LGR5, EpCAM (CD326), ALDH1, CDCP1, CXCR4, CC188 (Hsu et al., 2013; Pitule et al., 2014) and ephrin type B receptor 2 (EphB2) (Rowehl et al., 2014), although many of these markers are also expressed in normal colonic stem cells (i.e. LGR5, ALDH1, or CD29), complicating the distinction between CSCs and normal stem cells. From present knowledge, CRC MetSCs can be found among the cell population with a high expression of Wnt targets LGR5^+^ and EphB2^+^ (de Sousa e Melo et al., 2017; Jung et al., 2011; Kemper et al., 2012) co-expressing EpCAM, CD133, CXCR4 and CD26 markers. EpCAM, CD133 and CXCR4 are enriched in a metastatic cell population with an auto fluorescent subcellular compartment (Miranda-Lorenzo et al., 2014), and a CD26^+^/CD133^+^/CD44^+^ CSC population was capable of metastasizing when transplanted in mice (Pang et al., 2010). Most of these markers are co-expressed in the primary tumor; so, it is expected that a particular marker combination may be used to identify MetSCs in CRC. Intriguingly, a CD26^+^ circulating tumor cell (CTC) population that is CD44^+^ and CD66c^+^ but EpCAM^−^ and CD133^−^ is an independent prognostic factor for CRC recurrence (Lieto et al., 2015).

As the number of cells presenting CSC features that can be obtained from surgical samples is scarce (Oskarsson et al., 2014; de Sousa e Melo et al., 2017; Varela-Calviño and Cordero, 2015; James et al., 2015) and CTCs from liquid biopsies are highly heterogeneous (Khoja et al., 2015; Acosta et al., 2016), several studies have previously explored the possibility of using established cell lines. In spite of their inherent genetic instability during long-term passaging, CTCs are a valid option (Rowehl et al., 2014; Dotse and Bian, 2016), as they are able to sustain different cell subsets with CSC features (Zimmerer et al., 2013) and with the inherent heterogeneity observed in cancer populations (Driessens et al., 2012; Pitule et al., 2014; Lieto et al., 2015; Zimmerer et al., 2013). The aim of this study was to test candidate CSC and MetSC markers in a panel of eight cell lines obtained from primary or metastatic tumors of patients in different disease stages [so that epithelial-mesenchymal transition (EMT) markers were also used]. The results would provide a functional characterization of specific cell subsets found in clinical specimens (Lieto et al., 2015; Cheung et al., 2017) and the possibility of using these chemo-resistant cells as targets for the development of new therapies.

## RESULTS

### Expression of stem cell, CSC and EMT markers in CRC cell lines

**Fig. 1.**
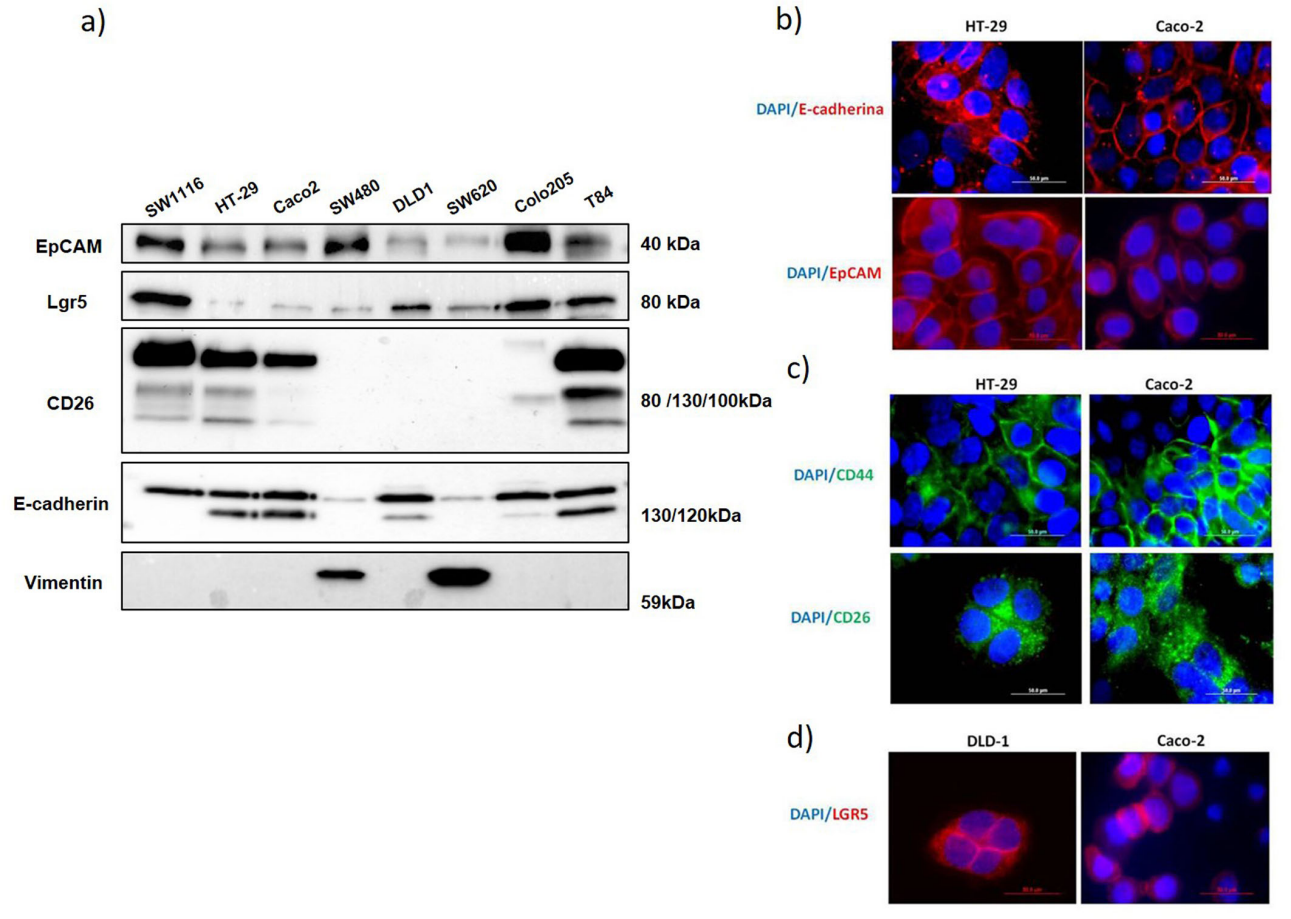
**Expression of different markers in the eight human colon cancer cell lines analyzed.** (A) Western blot analysis of EpCAM, LGR5, CD26, E-cadherin and vimentin expression in total cell extracts from the eight cell lines (20 μg of protein in each line). Data shown are representative of three experiments. (B) E-cadherin and EpCAM expression analysis by immunofluorescence in HT-29 and Caco-2 cells. (C) CD44 and CD26 expression analysis by immunofluorescence in HT-29 and Caco-2 cells. (D) LGR5 expression analysis by immunofluorescence in DLD-1 and Caco-2 cells. Nuclei were stained with DAPI. Scale bars: 50 μm.

The stem cell, CSC and EMT marker expression profile in eight CRC cell lines was analyzed by flow cytometry (Table 1), western blotting (Fig. 1A) and immunofluorescence (Fig. 1B–D). Cytometry results (Table 1) showed that cell lines SW1116, SW480 and SW620 presented the lowest E-cadherin expression. In western blotting (Fig. 1A), the mature form of E-cadherin (120 kDa) was detectable in DLD-1, HT-29, Caco-2, COLO205 and T84 but not in SW1116, SW480 and SW620, supporting the flow cytometry results. An E-cadherin high MW band (130 kDa), possibly corresponding to the inactive precursor (Khoja et al., 2015), was detected in all tested cell lines, although the expression was very low in SW480 and SW620. On the contrary, vimentin was only expressed in SW480 and SW620 (Fig. 1A), as expected for a mesenchymal stage and according to the expression observed for E-cadherin.

**Table 1.**
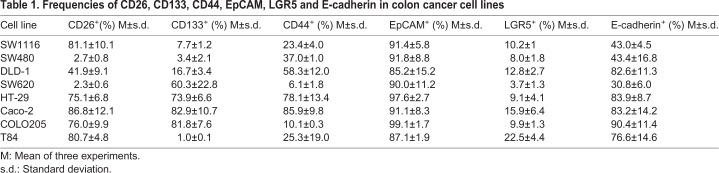
**Frequencies of CD26, CD133, CD44, EpCAM, LGR5 and E-cadherin in colon cancer cell lines**

A very high frequency of EpCAM^+^ cells was found in all cell lines (Table 1) and a band of 40 kDa was observed upon western blotting for all cell lines (Fig. 1A), suggesting that these cell lines were fully differentiated carcinoma cells. On the contrary, all cell lines showed a percentage of stem cells from intestinal origin as indicated by LGR5 expression, with the highest frequency in T84 cells (22.5%).

Cell lines with the lowest E-cadherin-expression showed low frequencies of CD44 (SW1116 and SW620), CD26 (SW480 and SW620), LGR5 (SW620), and CD133 (SW1116 and SW480) (Table 1) by flow cytometry. However, the expression of these CSC markers was very heterogeneous in the other cell lines, CD133 and CD44 being the most variable (between 1% in T84 and 82.9% in Caco-2 and between 10.1% in COLO205 and 85.9% in Caco-2, respectively). In the case of CD26, all the cell lines showed a high expression, except SW480, SW620 and DLD-1 (intermediate frequency of positive cells). In DLD-1 and COLO205, there was a lack of correlation between western blotting (Fig. 1A) and flow cytometry data (Table 1 and Fig. S1), which should be further investigated.

We also analyzed the autofluorescence phenotype of cell lines (exclusive of epithelial CSC), which could be enhanced using riboflavin (Miranda-Lorenzo et al., 2014). All the analyzed cell lines had a very small subpopulation of CSCs (Table S2).

Immunofluorescence (Fig. 1B–D) showed that the E-cadherin and EpCAM staining distributions were very similar and corresponded to those of proteins related to cell–cell interaction. The distributions of CD44, CD26 and LGR5 staining were more diffused, although they were plasma membrane proteins. However, CD26 staining showed a trend for polarization that LGR5 lacked.

### Phenotypic characterization of subsets in the cell lines

Current knowledge of normal and tumor tissues indicates that CSCs are rarely defined by a single marker but by a combination of multiple molecular markers. On the other hand, several studies have linked high surface expression of some of these markers with the tumor degree of differentiation, depth of invasion, clinical stage and metastatic status in CRC (Acosta et al., 2016; Dotse and Bian, 2016; Ren et al., 2013; LaBarge and Bissell, 2008; Kojima et al., 2008; Horst et al., 2008). Therefore, we established all possible combinations between the markers and all possible combinations of subpopulations with high intensity expression of markers.

For double positive subsets (Table S3), we observed high frequencies of almost all the markers in HT-29 and Caco-2 and very low frequencies in SW480 and SW620, as expected. The other cell lines showed different expressions depending on the analyzed subset. We observed that almost all LGR5^+^ cells in the cell lines were EpCAM^+^ (Tables 1 and S3) and had higher frequency of CD26^+^/E-cadherin^+^ cells than of CD133^+^/E-cadherin^+^ cells.

Markers with high expression in the cell lines were also observed. Fig. S2 shows dot plots for LGR5 versus EpCAM and CD133 versus CD26 in COLO205 and Caco cell lines, as examples of the gating strategy. Cells with high expression of EpCAM were easily detected in COLO205 and Caco cell lines, and all the cell lines had EpCAM^high^/LGR5^+^ subsets (from 2.8% in SW620 to 11.9% in Caco-2, Table 2), although not all LGR5^+^ cells were EpCAM^high^. In the case of dot plots for CD133 versus CD26 (not studied in this context before) (Fig. S2B, Table 3), one cell line (Caco-2) showed large CD133^+^CD26^high^ and CD133^high^/CD26^high^ subsets (65.3% and 9.6%, Fig. S2B). Very small CD133/CD26 subsets with high expression of one or both markers were observed in seven out of the eight analyzed cell lines (Table 3). In addition, the cell lines had CD133^−^/CD26^high^ subpopulations, except the mesenchymal cell lines (Table S4).

**Table 2.**
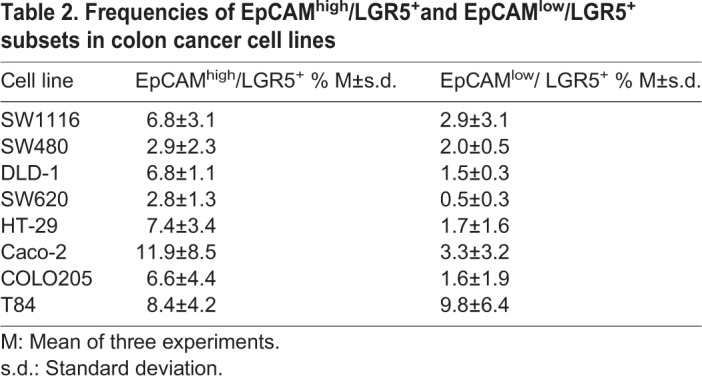
**Frequencies of EpCAM^high^/LGR5^+^and EpCAM^low^/LGR5^+^ subsets in colon cancer cell lines**

**Table 3.**
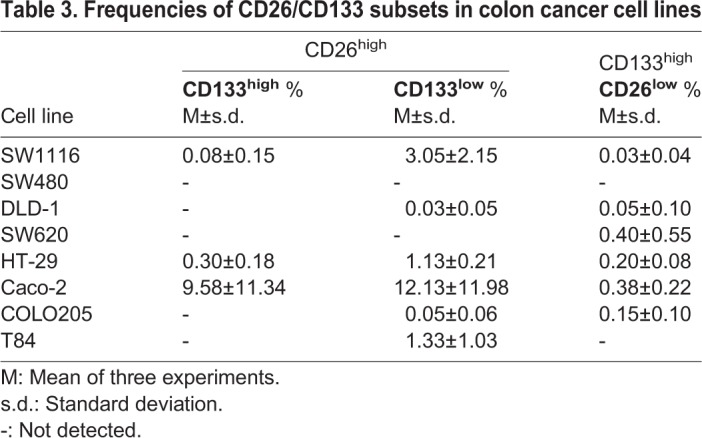
**Frequencies of CD26/CD133 subsets in colon cancer cell lines**

We also looked at possible E-cadherin^high^ subsets. Most cell lines (SW1116, DLD-1, HT-29, Caco-2, COLO205 and T84) harbored CD26^high^/E-cadherin^high^, CD26^high^/E-cadherin^+^, or E-cadherin^high^/CD26^+^ subsets (Table S5), but SW480 and SW620 did not show any of these subsets. CD133^high^ associated to E-cadherin^high^ was practically non-existent, and CD133 staining was poor in E-cadherin^+^ cells (Table 1 and Table S6).

No subset with high expression of CD44 was observed (data not shown), but the cells from the CD133^high^/CD26^high^ subset in the Caco-2 cell line were CD44^−^ and E-cadherin^low^.

### Sphere formation in the CRC cell lines under investigation

Whether the cell lines which originated from both primary and metastatic tumors were able to form spheres was examined. All cell lines formed spheres in the first generation after 7 days of culture (Fig. S3), suggesting the presence of CSCs in all cell lines. However, there were appreciable differences in the structure and size of the spheres (Fig. S3 and Table S7). SW480, SW620 and COLO205 spheres showed similar morphology and were different from the other cell line spheres. Those three cell lines had a low expression of E-cadherin (120 kDa) (Fig. 1B) as well as a low frequency of CD44 (Table 1). A lack or low expression of E-cadherin and CD44 could lead to decreased cell-to-cell contacts and to the observed morphology. However, SW1116 cell line also showed similar marker expression.

The sphere cells were disaggregated, and cells reseeded to establish self-renewal capacity by formation of secondary spheres (Table S7). Only SW1116 did not form secondary spheres. All the other cell lines did form spheres in three serial passages: DLD-1 formed more spheres and T84 less spheres (data not shown). Efficiency for self-renewal was essentially maintained through passages in all cell lines, T84 cells being the most efficient and SW620 the least efficient (Table S7).

### Expression of stem cell, CSC and EMT markers in cells derived from spheres

The spheres developed in 7-day cultures were disaggregated, and stem cell, CSC and EMT markers were analyzed by flow cytometry in sphere-derived cells (_sph_) (Table 4).

**Table 4.**
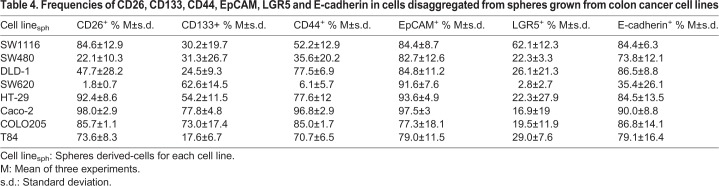
**Frequencies of CD26, CD133, CD44, EpCAM, LGR5 and E-cadherin in cells disaggregated from spheres grown from colon cancer cell lines**

Sphere-derived cells from all cell lines showed high frequencies of E-cadherin expression, except SW620 (Table 4). As expected for proliferating cells in epithelial state, E-cadherin frequencies were much higher in SW1116_sph_ and SW480_sph_ than those in their respective cell lines (Table 1). However, this was not the case in SW620_sph_ and T84_sph_, where the frequencies of E-cadherin^+^ were similar to the original cell line (being a minority in SW620).

**Fig. 2.**
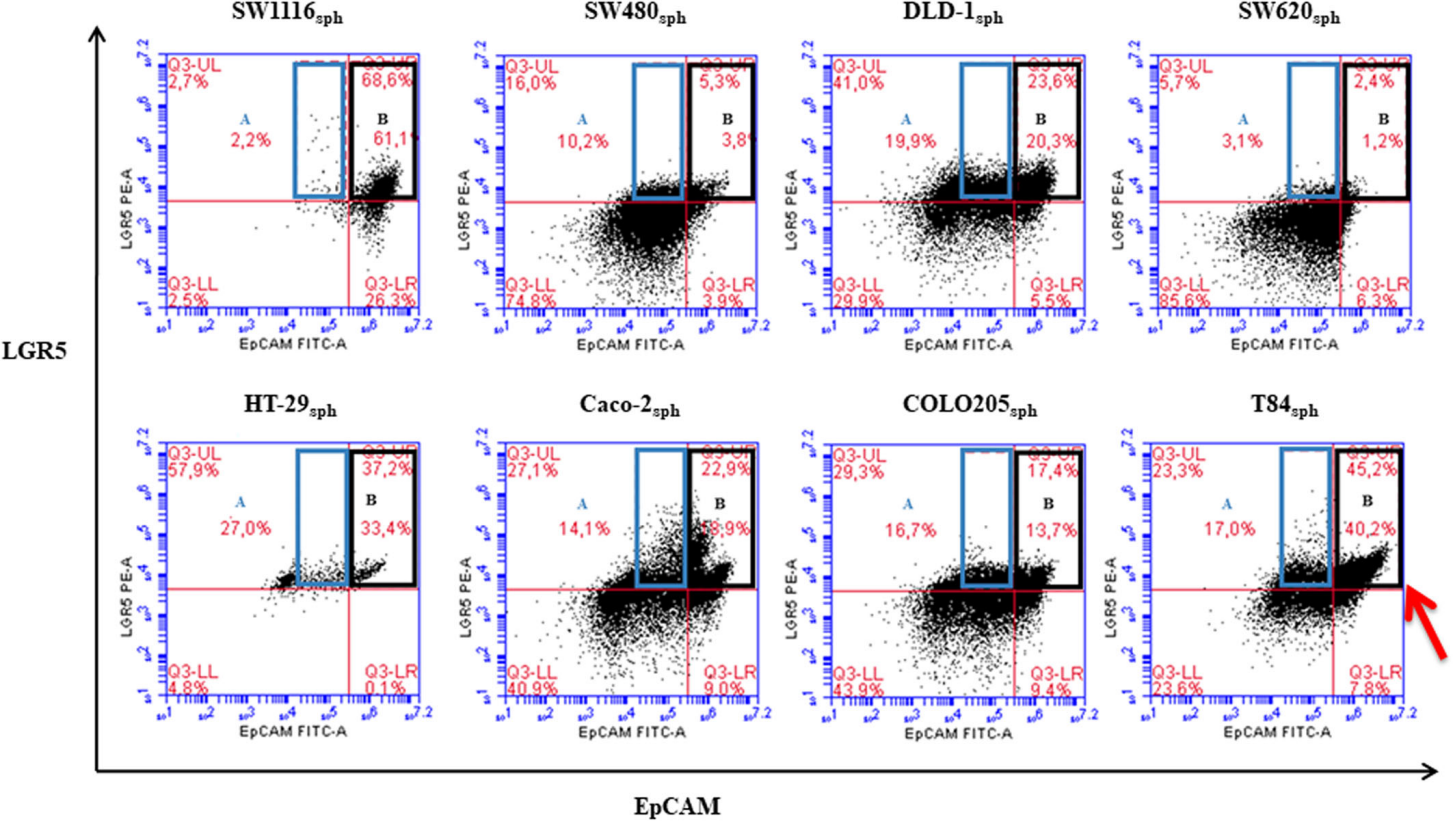
**Flow cytometry analysis of LGR5/EpCAM in sphere-derived cells.** T84_sph_ showed a subset with LGR5 high expression (LGR5^high^, red arrow). LGR5^+^/EpCAM^low^ region A, LGR5^+^/EpCAM^high^ region B.

More than half of SW1116_sph_ (62%) was LGR5^+^, with approximately 20% of positivity for this marker in the rest of cell lines_sph,_ except SW620_sph_, where only a minor subset (2.8%) was LGR5^+^ (Table 4). Sphere-derived cells showed enhanced frequencies (two- to three-fold) of LGR5 positivity compared to the original cell lines (Tables 4 and 1). All the cell lines_sph_ showed high frequencies of EpCAM^+^ (Table 4). SW1116_sph_, with high frequencies of LGR5^+^, also showed a high frequency (around a 50%) of the LGR5^+^/EpCAM^high^ subset (Table S8, Fig. 2, region B of dot plots). On the contrary, more EpCAM^low^ were found in SW480_sph_, SW620_sph_ and COLO205_sph_ (SW480_sph_ and SW620_sph_ showing the lowest frequencies of LGR^+^). In addition, the large subset of LGR5^+^/EpCAM^high^ in T84_sph_ showed some LGR5^high^ cells (Fig. 2, red arrow), which has not been described before.

The frequencies of CD133, CD44 and CD26 in cell lines_sph_ resembled those of the original lines, although there were some changes: CD133^+^ frequencies in T84_sph_ were higher than those in the original cell line (17%), whereas HT-29_sph_ and COLO205_sph_ showed lower CD133 frequencies than those in the lines of origin (Fig. S4). CD44 was also found at high frequencies in cell lines_sph_, except in SW620_sph_ (Table 4). Frequencies were also low in SW480_sph_ compared to the sphere-derived cells from the other cell lines. CD26 showed particularly higher frequencies in SW480_sph_ than those in the original cell line, despite being strikingly similar to the original cell lines in the other cases (Tables 1 and 4).

Very interestingly, CD133, CD44 and CD26 hardly correlated (Fig. S4). Intriguingly, although the same cell lines continued to show a CD26^high^ population in sphere-derived cells, the frequency was quite a lot lower in Caco-2_sph_ and much higher in T84_sph_ than those in the original cell lines (Tables S9 and S4). To note, the latter are the ones with EpCAM^high^/LGR5^high^. The CD133^high^/CD26^high^ subsets were very small in cell lines_sph_ but more detectable than in the original cell lines (Table S10). However, the subsets of CD133^high^/CD26^−^ cells in some cell lines (e.g. SW620_sph_ with 4.2%) or in the other combinations (Table S10) showed higher frequencies than those in the original cell lines.

### Relationship between CSC and EMT markers in cells derived from spheres

All cell lines_sph_ showed some E-cadherin^−^ cells and E-cadherin^+^ subsets corresponding to mesenchymal and epithelial cells, respectively. The subsets of E-cadherin^−^ cells in all cell lines_sph_ had a higher percentage of small size cells than that of the subsets of E-cadherin^+^ cells (e.g. DLD-1_sph_ in Fig. 5). We analyzed if CSC markers were differentially expressed in both E-cadherin^−^ and E-cadherin^+^ sphere-derived cells.

### Markers in E-cadherin^−^ sphere-derived cells

Most but not all E-cadherin^−^ cells are also CD133^−^, CD26^−^, or CD44^−^ (e.g. CD133 in Fig. 5). A common characteristic in these E-cadherin^−^ cells was the presence of one or more subsets expressing only one marker in different combinations in all cell lines_sph_, and co-expression of CD133/CD26 was not found in five of them. SW1116_sph_ was CD133^+^, CD26^+^ and CD44^+^, and had a few CD26^+^/CD44^+^ cells (2.3%); SW480_sph_ and SW620_sph_ were totally CD26^−^, with a high percentage of CD133^+^ and a low percentage of CD44^+^ or CD133^+^/CD44^+^; DLD-1_sph_ had small subsets of only CD26^+^, CD44^+^ and CD133^+^ cells; HT-29_sph_ had a small subset of CD133^+^/CD26^+^/CD44^+^, CD44^+^/CD26^+^, or only CD44^+^ cells; Caco-2_sph_ had a large CD133^+^/CD26^+^/CD44^+^ and CD26^+^/CD44^+^ subsets and a small CD26^+^ or CD44^+^ subset; COLO205_sph_ had a large CD26^+^ subset and a small CD133^+^/CD26^+^ subset; T84_sph_ was mostly CD133^−^, with a high CD26^+^ percentage and a low percentage of CD44^+^ or CD26^+^/CD44^+^.

### Markers in E-cadherin^+^ sphere-derived cells

Although still having small cells, the majority of the cells in the E cadherin^+^ subset were large, and almost all large cells were in this subset (Fig. S5). The frequency of the CD26^+^/CD44^+^ subset increased independently of the expression of CD133, although there were still cells only CD26^+^ or CD44^+^ (data similar to Fig. S4). Moreover, this CD26^+^/CD44^+^ subset correlated with the stage of origin of the cell line (larger subsets in cell lines from advanced stage tumors, data not shown), irrespective of the presence of CD133.

A correlation between E-cadherin and CD26 was observed in most cell lines_sph_. A population of E-cadherin^high^ cells was observed in all cell lines_sph_ (Fig. S6, regions A and B), except in SW620_sph_, and these cells were mainly CD26^high^ (Fig. S6, region B, and Table S11). A similar profile was found many times for CD44 (data not shown). However, in the case of CD133, although some E-cadherin^high^ cells showed CD133 staining (Table S6), most CD133^+^ cells were E-cadherin^−^ (Table 4 and Table S3).

**Fig. 3.**
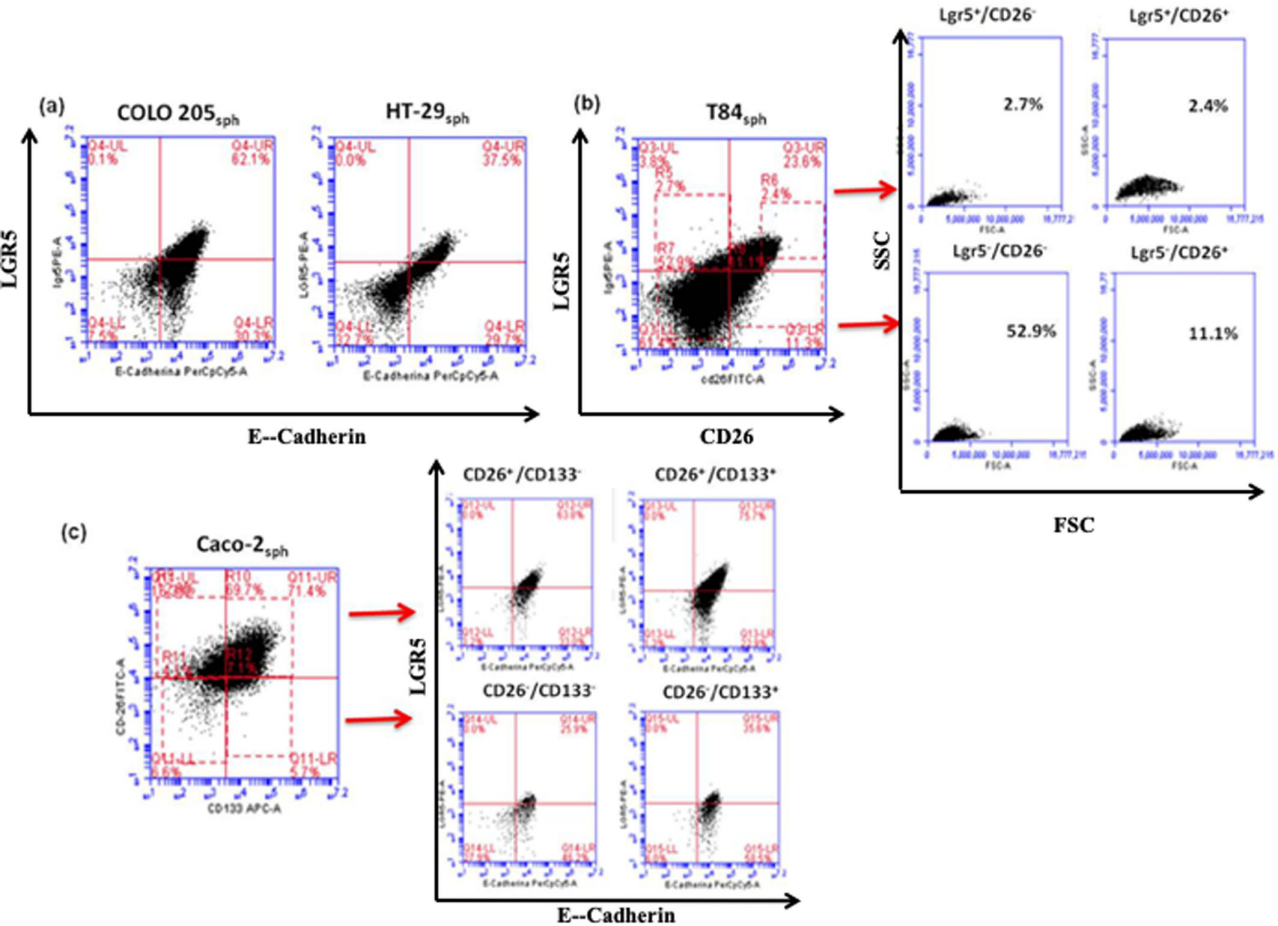
**Flow cytometry analysis of LGR5/E-cadherin, LGR5/CD26 and CD26/CD133 in sphere-derived cells.** (A) Two representative dot plots of LGR5 versus E-cadherin expression in COLO205_sph_ and HT-29s_ph_. (B) LGR5 versus CD26 expression in T84_sph_. CD26^high^/LGR5^+^cells are marked in R6 region in UR quadrant. Physical gatings (FCS versus SSC) of the four quadrants of T84_sph_ are shown. (C) Caco-2_sph_ representative dot plots for CD26 versus CD133 and, on the right, LGR5 versus E-cadherin dot plots of the four regions gated on the left CD26/CD133 dot plot.

Interestingly, positivity for E-cadherin and LGR5 and their fluorescence intensity were strongly correlated (Fig. 3), and no LGR5^+^ cells were E-cadherin^−^. These cells were also more complex in morphology (SSC axes in Fig. 3B). As shown in Fig. 3B (region 6 in the UR quadrant) and from other results not shown, we confirmed that when CD26^high^ cells were present, they were LGR5^+^. However, there were some LGR5^+^ cells without CD26 and CD133 markers (e.g. Caco-2_sph_ in Fig. 3C).

Therefore, as LGR5 and E-cadherin expression correlate in sphere cells (Fig. 3A) – as well as LGR5 with EpCAM (Fig. 2) and with CD26 (Fig. 3B), and also E-cadherin with CD26 (Fig. S6) but not with CD133 (Fig. 3C) – it can be deduced that, in sphere derived cells from lines of advanced tumor stage, the E-cadherin^+^/LGR5^+^/EpCAM^high^ cells are also CD26^high^. Some of these E-cadherin^+^/LGR5^+^/EpCAM^high^ /CD26^high.^, but not all, were CD133^+^ or CD133^high^ (Fig. 3C), and most were CD44^+^ (Fig. S4).

### Clustering of markers in sphere-derived cells

**Fig. 4.**
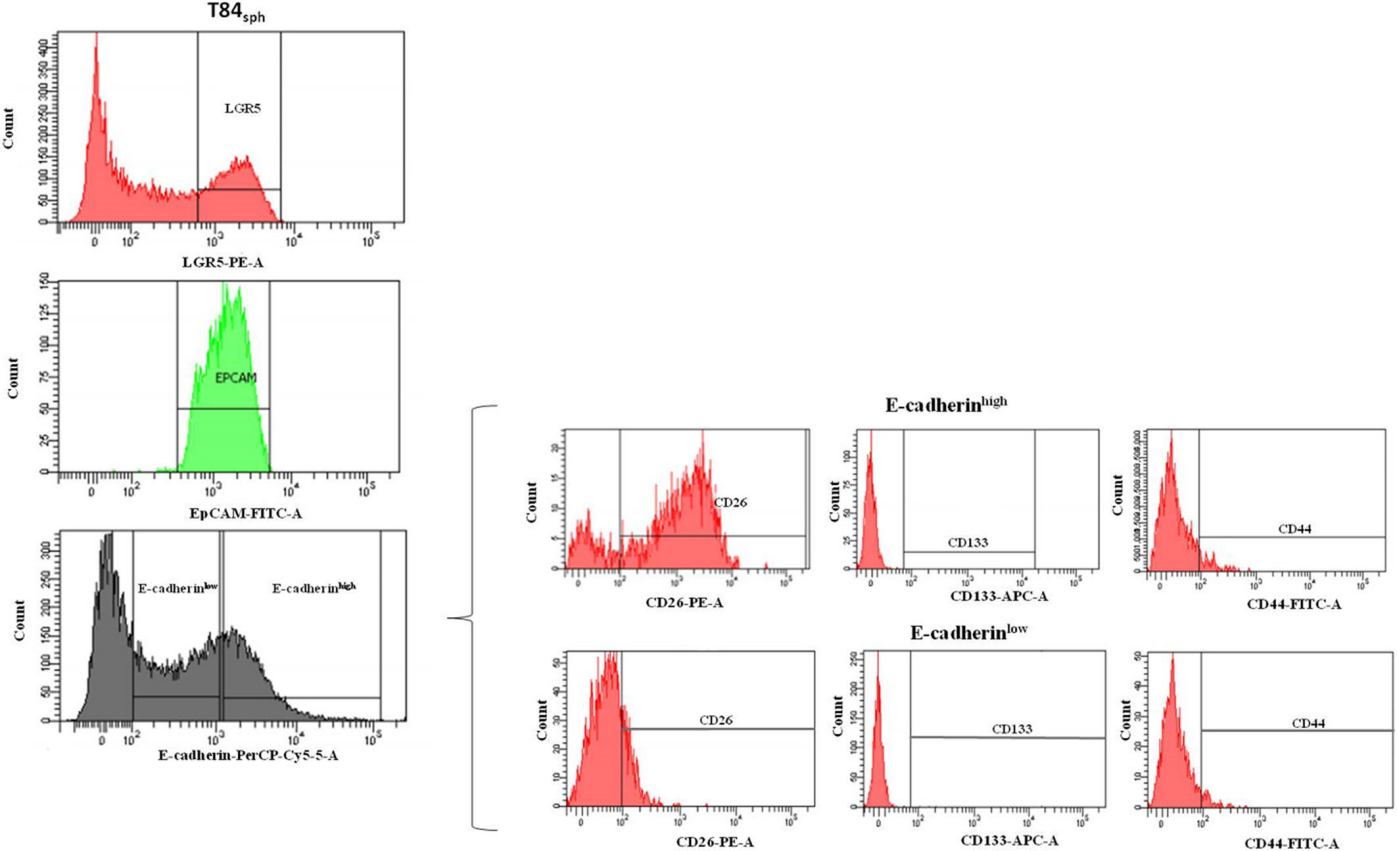
**Sorting strategy of T84_sph_.** T84_sph_ LGR5^+^ (top histogram) and EpCAM^high^ (middle histogram) were sorted into E-cadherin^low^ and E-cadherin^high^ subsets (bottom histogram). E-cadherin^low^ and E-cadherin^high^ subsets (on the right) were analyzed for CD26, CD133 and CD44 expression.

To confirm the above finding, LGR5^+^/EpCAM^high^ subsets from T84_sph_ were sorted into E-cadherin^low^ and E-cadherin^high^ subsets, which were analyzed for CD26, CD44 and CD133 expression (Fig. 4). Both subsets showed low expression of CD133 and CD44. The E-cadherin^high^ subset was mostly CD26^+^ (82%), while the E-cadherin^low^ subset was only 20% CD26^+^.

## DISCUSSION

We show for the first time that LGR5^+^, E-cadherin^high^, EpCAM^high^ and CD26^high^ are frequently associated in sphere-derived cells, that CD133 seems to be related to a different germinal line, and that cell division affects the expression of all markers, including that of E-cadherin*.* These results are highlighted with the recent report that has shown that LGR5^+^ cells are more important for the process of metastasis than for primary tumor growth (de Sousa e Melo et al., 2017).

Circulating human CD133^+^/CD26^+^/CD44^+^ cells but not CD133^+^/CD26^−^/CD44^+^ cells have been detected in the portal vein of mice at week 6 after cecal wall injection, demonstrating the invasion of CD26^+^ cells into the circulation of orthotopically implanted mice, leading to the development of liver metastasis (Pang et al., 2010). Interestingly, E-cadherin expression was down-regulated in CD133^+^/CD26^+^ cells from primary CRC tumors compared to CD133^+^/CD26^−^ cells, with the concomitant upregulation of N-cadherin, the E-cadherin repressor slug, as well as other EMT markers, such as twist, fibronectin and vimentin (Pang et al., 2010; Cheung et al., 2017). Here, we show that, in most analyzed cell lines, CD133 and CD26 are hardly expressed together and CD133^−^/CD26^+^ sphere cells (perhaps CSCs) are a major population. Although the subset E-cadherin^low^ or^−^/CD133^+^/CD26^+^ could be found in all lines and also in sphere-derived cells, the E-cadherin^high^/CD133^−^/CD26^+^ subset was particularly large in cell lines from advanced CRC stages. This result is consistent with the fact that in humans, the population isolated from the primary tumor comprising CD133^+^/CD44^+^/CD26^+^ cells (and E-cadherin^low^ or E-cadherin^−^) is not the only CSC population present in the tumor biopsies (Pang et al., 2010).

Several studies linked CD133^high^ expression with a high risk of metastasis in CRC patients (LaBarge and Bissell, 2008; Kojima et al., 2008; Horst et al., 2008; Ong et al., 2010; Gallmeier et al., 2011), but the effective value of CD133 as a CSC biomarker is unclear, because, as observed in the SW620 colon cell line, sorted CD133^+^ and CD133^−^ subsets can undergo conversion between the two subsets (Hsu et al., 2013; LaBarge and Bissell, 2008; Kojima et al., 2008).

E-cadherin was used as a control of EMT. CSCs exist both in epithelial and mesenchymal states (Liu et al., 2014a,b), but EMT favors migration of cancer cells while inhibiting cell proliferation. Thus, MetSCs should be found in the epithelial state in the primary tumor, in the mesenchymal state in the peripheral blood (Oskarsson et al., 2014; Zimmerer et al., 2013), and in the epithelial state in the host organ. We assume that the loss of E-cadherin expression in sphere-derived cells (spheres were obtained over a 7-day period) is because they are newborn proliferating cells (that is, epithelial cells but lacking E-cadherin), rather than mesenchymal cells. However, the discrimination between sphere-derived small cells losing E-cadherin expression and mesenchymal cells proliferating should be a priority of further research because these small cells, which completely lost EpCAM and LGR5 expression (recovered as they enlarged), were found in every passage of sphere-derived cell cultures, thus, data of LGR5 positivity could underestimate the frequency of intestinal CSCs in this work and *in vivo*. Interestingly, CD133, CD26 and CD44 expression remained in some of these small cells. The positivity for one or more of these markers was useful to identify each cell line, suggesting the presence of different lineages (e.g. E-cadherin^−^/CD133^+^ was the origin of the CD133^high^ subsets in the cell lines). In a very important study that described the stemness of spheroid-derived stem-like colon cancer cells from lines, the cells used in this work, these markers were not tested (Han et al., 2013).

EpCAM (CD326) overexpression is an early event during cancer progression in some types of tumors such as prostate and lung cancer, as well as in CRC (Miranda-Lorenzo et al., 2014; Pitule et al., 2014; Rowehl et al., 2014; Liu et al., 2014a,b). EpCAM appears in 85% of colorectal carcinomas, it can inhibit differentiation and promote proliferation (Hsu et al., 2013), and it is used to isolate CTCs in liquid biopsies (Dotse and Bian, 2016).

Although the presence of EpCAM^high^/CD44^+^ cells correlated with the degree of differentiation, depth of invasion, clinical stage and metastatic status in CRC (Liu et al., 2014a,b) and gastric cancers (Han et al., 2011), because of the plasticity and the not completely known role of their isoforms, CD44 expression does not seem to be an appropriate marker of MetSCs (Hsu et al., 2013; Pitule et al., 2014; Rowehl et al., 2014; Qiu et al., 2015; Nagano and Saya, 2004). However, in E-cadherin expressing cells, we observed that the frequency of the CD26^+^/CD44^+^ subset increased independently of the expression of CD133.

LGR5 is a well-characterized marker of intestinal and colon stem cells (de Sousa e Melo et al., 2017; Kemper et al., 2012; Osawa et al., 2016; Zhang et al., 2016; Basu et al., 2016). We observed that sphere-derived cells showed a high frequency of the LGR5^+^/EpCAM^high^ subset in many cell lines. This subset can represent clonogenic CSCs that proliferate originating the sphere (de Sousa e Melo et al., 2017; Zimmerer et al., 2013). However, in some cell lines, the frequency of LGR5^+^/EpCAM^low^ cells was high and there were very low frequencies of EpCAM^high^ found in SW480_sph_, SW620_sp__h_ and COLO205_sph_. Interestingly, a small LGR5^+^/EpCAM^−^ subset was detected in all lines_sph_, probably representing the CD26^+^/CD44^+^/CD66c^+^ but EpCAM(CD326)^−^ and CD133^−^ CTC population that was an independent prognostic factor for CRC recurrence (Lieto et al., 2015).

Based on the fact that there are immune helper T cells with a defined CD26^high^ phenotype (Krakauer et al., 2006), we tested if a similar subset with high staining of CD26 was present in CRC cell lines. Our data showed that the EpCAM^high^/LGR5^+^ subset had high expression of E-cadherin and CD26, with variable frequency among the cell lines. The subset was mostly CD44^+^, and CD133^+^ or CD133^−^, but there were also cells only CD26^+^. The frequency of this E-cadherin^+^/LGR5^+^/EpCAM^high^/CD26^high^ lineage resembled that of the auto-fluorescent CSCs (Miranda-Lorenzo et al., 2014).

In addition, cell lines had cells only CD133^+^, particularly cell lines from early stages. The CD133^high^/CD26^high^ and CD133^high^/CD26^+^ subsets present in the cell lines (both CD44^−^ and E-cadherin^low^, unlike the CD133^+^/CD26^high^ subset) were not found in sphere-derived cells, suggesting additional clonogenic subsets that might be less related to the metastatic process (de Sousa e Melo et al., 2017).

In a previous study where the effect of chemotherapeutic adjuvants 5-fluorouracil, vinblastine, oxaliplatin, methotrexate, or irinotecan was studied on cell lines and in orthotropic tumor cells (Cutler et al., 2015), chemotherapy agents led to the loss of CXCR4^+^, another candidate marker in epithelial cancer cells (Miranda-Lorenzo et al., 2014; Jung et al., 2011; Rowehl et al., 2014; Cutler et al., 2015; Liao et al., 2010; Davies et al., 2015), in combination with CD133^+^ (CXCR4^+^/CD133^+^), and to the enrichment of CD26^+^/CD44^+^ cells, in agreement with our results, although our data show that these chemotherapy agents are not necessary to enhance CD26 and CD44 expression, as Cutler et al. suggest (Cutler et al., 2015). All in all, these data are supporting the role of CD26 in MetSCs (Cheung et al., 2017; Liao et al., 2010; Davies et al., 2015; Lam et al., 2014; Nishikawa et al., 2015; Collura et al., 2013; Gemei et al., 2013; Grunt et al., 2015) and the presence of at least two lineages of CSCs (Basu et al., 2016).

CD26 is related to some extent to the CXCR4/SDF-1 axis, because SDF-1 (CXCL-12) is a substrate of CD26/DPP4 enzymatic activity. However, most of the markers studied, including CD26, are related to cell–cell adhesion directly (as E-cadherin or EpCAM) or indirectly through the ECM. CD26 is known to associate with ADA, fibronectin and collagen (Cheng et al., 2003; Ghersi et al., 2002; Naim et al., 1999), and its binding to proteoglycans versican and glypican-3 has been suggested (Havre et al., 2013; Khurana et al., 2013). CD44 is a receptor for hyaluronic acid (Liu et al., 2014a,b; Han et al., 2011). Nevertheless, whereas CD44 has functions only in cell adhesion and signaling, CD26 also has DPP4 activity in the intestinal brush border of normal mucosa with a role in protein digestion (Naim et al., 1999). To note, most primary tumors lose CD26 expression, and this may explain the difference between CD44 and CD26 staining of cell lines, although both can be cleaved from the cell surface (Nagano and Saya, 2004; Cordero et al., 2009). In fact, we demonstrated recently that soluble CD26 levels (sCD26) were a much better serum marker for the detection of CRC metastasis or tumor recurrence compared to other markers in clinical use, such as CEA, CA-19.9, or CA-72.4 (De Chiara et al., 2014). At the same time, a relationship between the presence of CD26^+^ cells, detected by immunohistochemistry in primary CRC tumor biopsies, and the prognosis of metastasis has been demonstrated (Grunt et al., 2015), as well as between the presence of CD26^+^/CD44^+^ and EpCAM^−^/CD133^−^ CTC population, detected in liquid biopsy, and the prognosis of colorectal cancer recurrence (Lieto et al., 2015). It remains to be elucidated if these cells or the LGR5^high^/E-cadherin^high^/EpCAM^high^/CD26^high^ cells described here cause metastasis in mice as the CD26^+^/CD133^+^/E-cadherin^low^ CSC population (Pang et al., 2010).

CD26 and/or dipeptidyl peptidase 4 inhibitors can prevent colon cancer and lung metastasis in animal models (Angevin et al., 2017; Jang et al., 2015; Femia et al., 2013; Yorifuji et al., 2016), although epidemiological studies in humans are not clear (Enz et al., 2019). There are also encouraging results regarding anti-CD26 Ab in mesothelioma, renal and urological tumors (Enz et al., 2019). Overall, studies investigating CD26^+^ subsets may ultimately contribute to the development of new treatment options for CRC (Cheung et al., 2017; LaBarge and Bissell, 2008; Han et al., 2013, 2011; Qiu et al., 2015; Osawa et al., 2016; Cutler et al., 2015; Khurana et al., 2013).

## MATERIALS AND METHODS

### Cell lines and culture conditions

Eight human colon cancer cell lines, SW1116, SW480, DLD-1, SW620, HT-29, Caco-2, COLO205 and T84 were obtained from the American Type Culture Collection (ATCC). SW1116, SW480, DLD-1, SW620, HT-29 and Caco-2 were cultured in DMEM media (Lonza) supplemented with 10% fetal bovine serum (FBS) (Sigma-Aldrich), 1% L-glutamine (Sigma-Aldrich) and 1% penicillin/streptomycin (Sigma-Aldrich). COLO205 was maintained in RPMI 1640 media (Lonza) supplemented with 10% fetal bovine serum (FBS), 1% L-glutamine and 1% penicillin/streptomycin. T84 was maintained in DMEM/Ham's F12 media (Lonza) supplemented with 10% FBS, 1% L-glutamine and 1% penicillin/streptomycin. Cells were grown at 37°C in a humidified atmosphere of 5% CO_2_.

Dukes' stage and tissue origin for each cell line is shown in Table S1.

### Sphere formation assay

Sphere formation media was composed of serum-free DMEM/F12 (1:1), 20 ng/ml epidermal grow factor (EGF; Calbiochem), 10 ng/ml basic fibroblast growth factor (bFGF; Calbiochem), 5 µg/ml insulin (Sigma-Aldrich), 1x B-27^®^ Supplement without Vitamin A (Gibco) and 1% penicillin/streptomycin (Sigma-Aldrich). For sphere suspension culture, cell lines grown in a two-dimensional monolayer were digested with trypsin (Lonza) and seeded at a density of 1×10^4^ cells/ml in serum-free medium (SFM), in 100 mm ultra-low attachment plates (Corning) at 37°C and in a humidified atmosphere of 5% CO_2_. The number and diameter of spheres were evaluated after 7 days. Medium was supplemented every alternate day to maintain proliferation and viability in all plates.

For serial passages, 7-day-old spheres were harvested and dissociated into single cells with trypsin. Dissociated cells were replaced in a new plate and cultured for 7 days. Efficiency of self-renewal was calculated from the number of cells formed in each passage from one single cell reseeded (100 cells/well).

### Antibodies

Antibodies used for western blot, immunofluorescence and flow cytometry were as follows: anti-CD26 (≠AF1180, R&D Systems), anti-CD26 (≠H00001803-D1 Novus Biologicals), anti-E-cadherin (≠610181, BD Biosciences), anti-vimentin (≠MA5-11883, Thermo Fisher Scientific Pierce), anti-EpCAM (≠2929, Cell Signaling Technology), anti-LGR5 (≠TA503316, OriGene Technologies), anti-CD133 (≠MAB4399, Millipore), anti-CD44-FITC (≠44F2, Immunostep), anti-CD133-APC (≠AC133 Miltenyi Biotech), anti-EpCAM-FITC (≠130-098-113, Miltenyi Biotech), anti-LGR5-PE (≠1030-100848, Miltenyi Biotech), anti-CD26-PE (≠26PE, Immunostep), anti-CD26-FITC (≠26F, Immunostep) and anti-E-cadherin-PerCP-Cy5.5 (≠563573, BD Biosciences).

Secondary antibodies used for western blot were horseradish peroxide (HRP)-conjugated antibodies (Sigma-Aldrich). Secondary antibodies used for immunofluorescence were goat anti-rabbit AlexaFluor^®^488 and goat anti-mouse AlexaFluor^®^594 (Thermo Fisher Scientific).

### Western blot analysis

Cells were lysed in RIPA buffer (50 mM Tris-HCl, pH 7.5; 150 mM NaCl; 1% NP-40; 0.5% sodium deoxycholate and 0.1% SDS), supplemented with protease inhibitor cocktail (Roche). Insoluble components were removed by centrifugation and protein concentrations were measured using the Bradford protein assay (Bio-Rad). Equal amounts of protein (20 µg) were separated by SDS-PAGE and transferred onto PVDF membranes (Immobilon-P, Millipore). Membranes were blocked with 5% non-fat dry milk in PBST (0.1% Tween-20) for 1 h and probed with the primary antibodies diluted in PBST/5% non-fat dry milk. After washing, membranes were incubated with secondary horseradish peroxide-conjugated antibodies. Protein signals were visualized with the Clarity Western ECL Substrate (Bio-Rad) according to the manufacturer's protocol. Images were acquired using a ChemiDoc XRS+ system (Bio-Rad). Quantitative image analysis was performed with Image lab software (Bio-Rad). Full-length blots of expression of different markers in the eight human colon cancer cell lines analyzed are showed in Fig S7.

### Immunofluorescence

Culture cells were grown in appropriate medium on glass coverslips until 70–80% confluence. Cells were fixed with 4% paraformaldehyde for 30 min at 37°C followed by permeabilization with 0.5% Triton X-100-phosphate buffered saline (PBS) for 3 min at room temperature. Cells were blocked with 2% bovine serum albumin in PBS for 30 min. Then cells were incubated with the appropriate primary antibodies for 1 h. After several washes, cells were incubated with appropriate fluorescent secondary antibodies for 45 min in the dark. Cell nuclei were stained with DAPI (1 µg/ml). The coverslips were mounted with MOWIOL on microscope slides and immunofluorescence was visualized using a fluorescence microscope (Olympus-BX51).

### Flow cytometry and cell sorting analysis

Human colon cancer cells derived from monolayer cultures and sphere-derived cells on day 7 after primary culture were adjusted to a final concentration of 10^6^ cells/ml. Cell suspensions were incubated for 10 min with blocking solution and with appropriate antibodies in the dark at 4°C for 30 min. Cells were then washed with PBS and analyzed by flow cytometry (BD Accuri™ C6, BD Biosciences). We repeated each characterization three times to validate the results observed. We followed the protocol of Miranda-Lorenzo et al. (2014) to acquire autofluorescent cells from cultured cells: 30 µM riboflavin (Sigma-Aldrich) was added to cultured cells. For FACS acquisition cells were incubated overnight at 37°C, centrifuged at 300 ***g*** for 5 min and cell pellets re-suspended in PBS. Autofluorescent cells were excited with 488–561 nm laser and selected as the intersection with filters 496/578. Propidium iodide (Sigma-Aldrich) was used for exclusion of dead cells. Cell sorting was performed in a FACSAria IIu analyzer (BD Biosciences) by using the PC FACSDiva software program (BD Biosciences).

### Gating strategies

Cells were gated on physical parameters (forward-scatter versus side scatter) to exclude dead or apoptotic cells, cell debris and aggregated cells. Single cells were gated on FSC-Area versus FSC-Height profile for excluding doublets.

An autofluorescence analysis was done with unstained cells and the background level was also determined for each fluorochrome. This helped us to evaluate the spillover of the different fluorochromes. Compensation controls were included for each fluorochrome.

Isotype controls were used to mark positive or negative staining. These marks were properly changed when any subset with high expressions (over the usual expression) of some CSC markers were observed in the different populations studied.

## Supplementary Material

Supplementary information
