# Supplementary material for: Surface expression marker profile in colon cancer cell lines and sphere-derived cells suggests complexity in CD26+ cancer stem cells subsets
Source: Biol Open. 2019 Jul 8;8(7):bio041673. doi: 10.1242/bio.041673 (PMC6679411; doi:10.1242/bio.041673)
Supplement: Supplementary information [file biolopen-8-041673-s1.pdf]

# SUPPLEMENTARY FIGURES

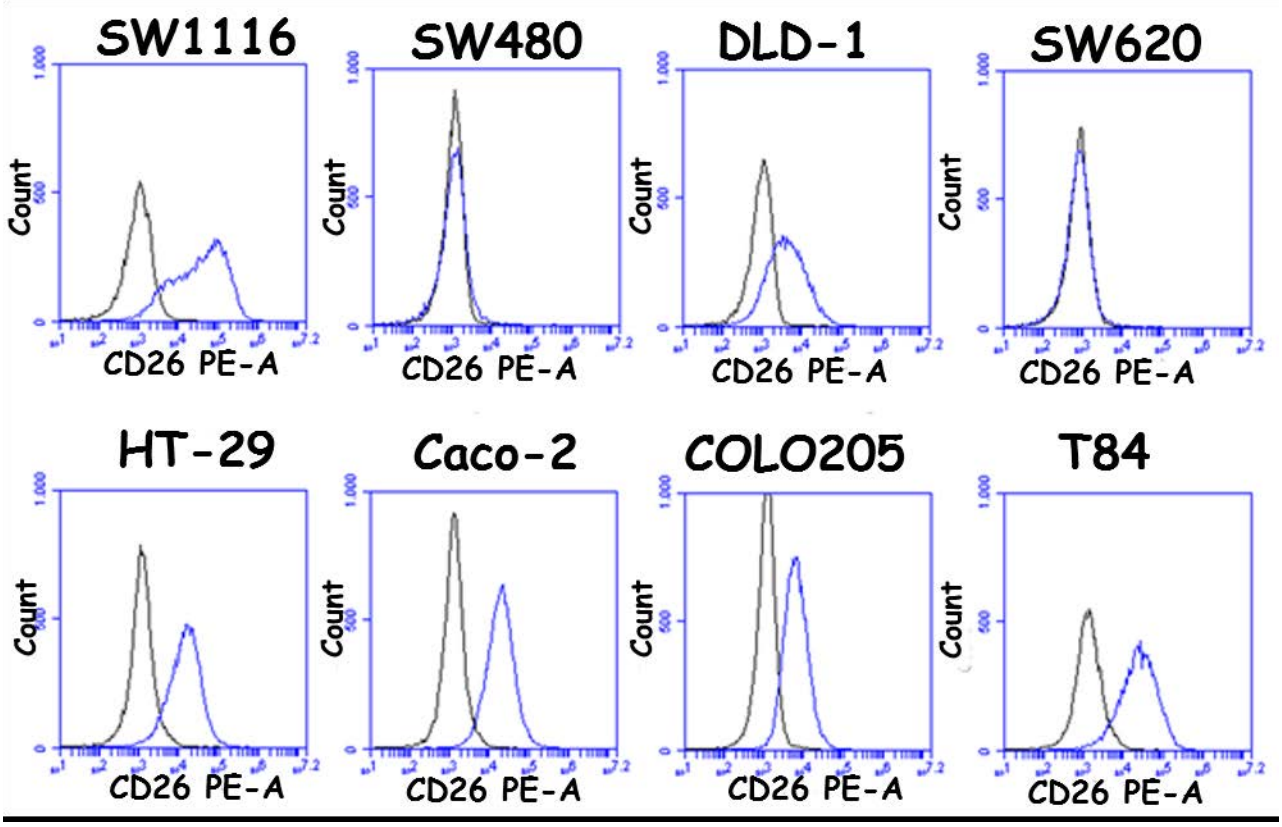


**Fig S1.** Flow cytometry analysis of CD26 expression in the eight human colon cancer cell lines analysed. Viable cells were gated and expression of CD26 was analysed (black line marks negative cells).

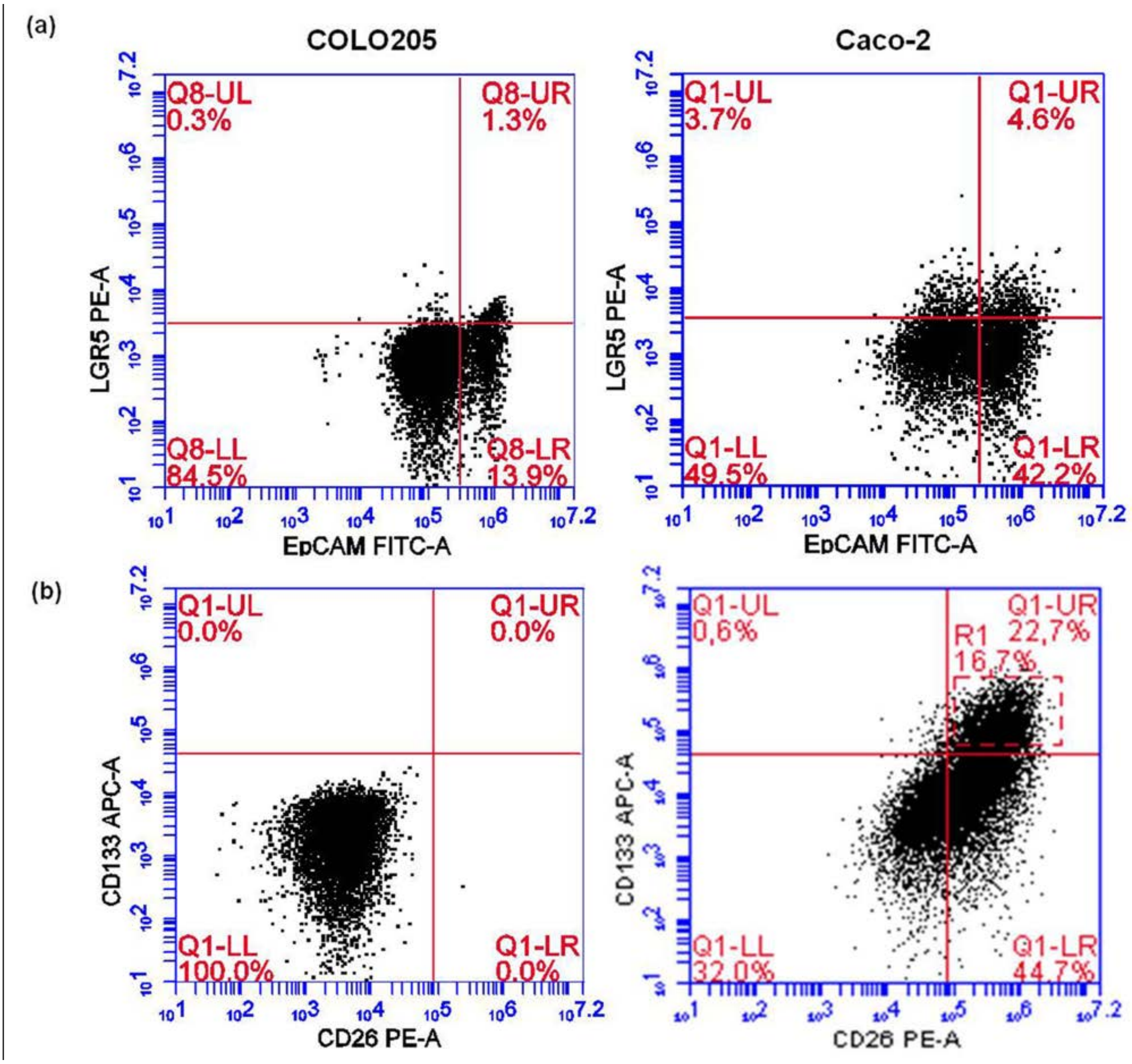


**Fig S2.** Flow cytometry analysis of candidate surface markers LGR5, EpCAM, CD133 and CD26. Representative dot plots are shown in COLO205 and Caco-2 cell lines. a) LGR5 *vs* EpCAM dot plots, b) CD133 *vs* CD26 dot plots. Quadrant R1 in Caco-2 dot plot (b) represents high expression CD133/CD26 subset.

Biology Open • Supplementary information

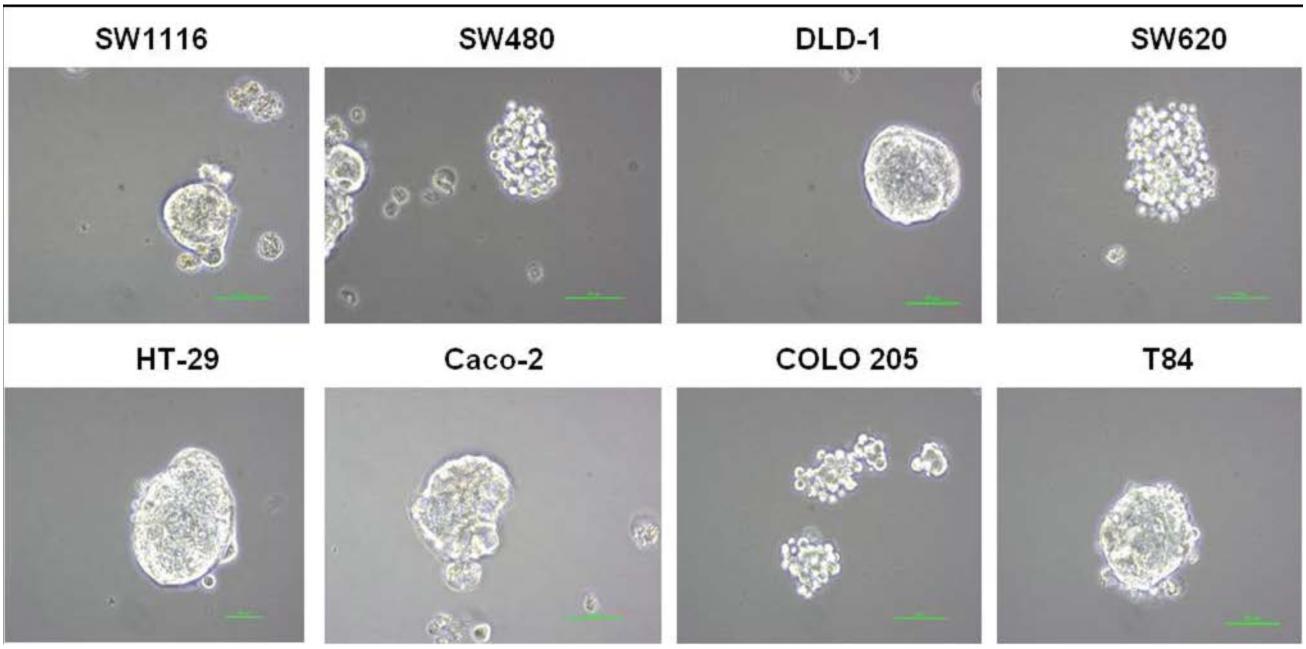


**Fig S3.** Morphology of spheres generated from colon cancer cells lines after 7 days in SFM. Representative pictures of spheres from SW1116, SW480, DLD-1, SW620, HT-29, Caco-2, COLO205 and T84 are shown. Scale bars: 50 μm.

Biology Open • Supplementary information

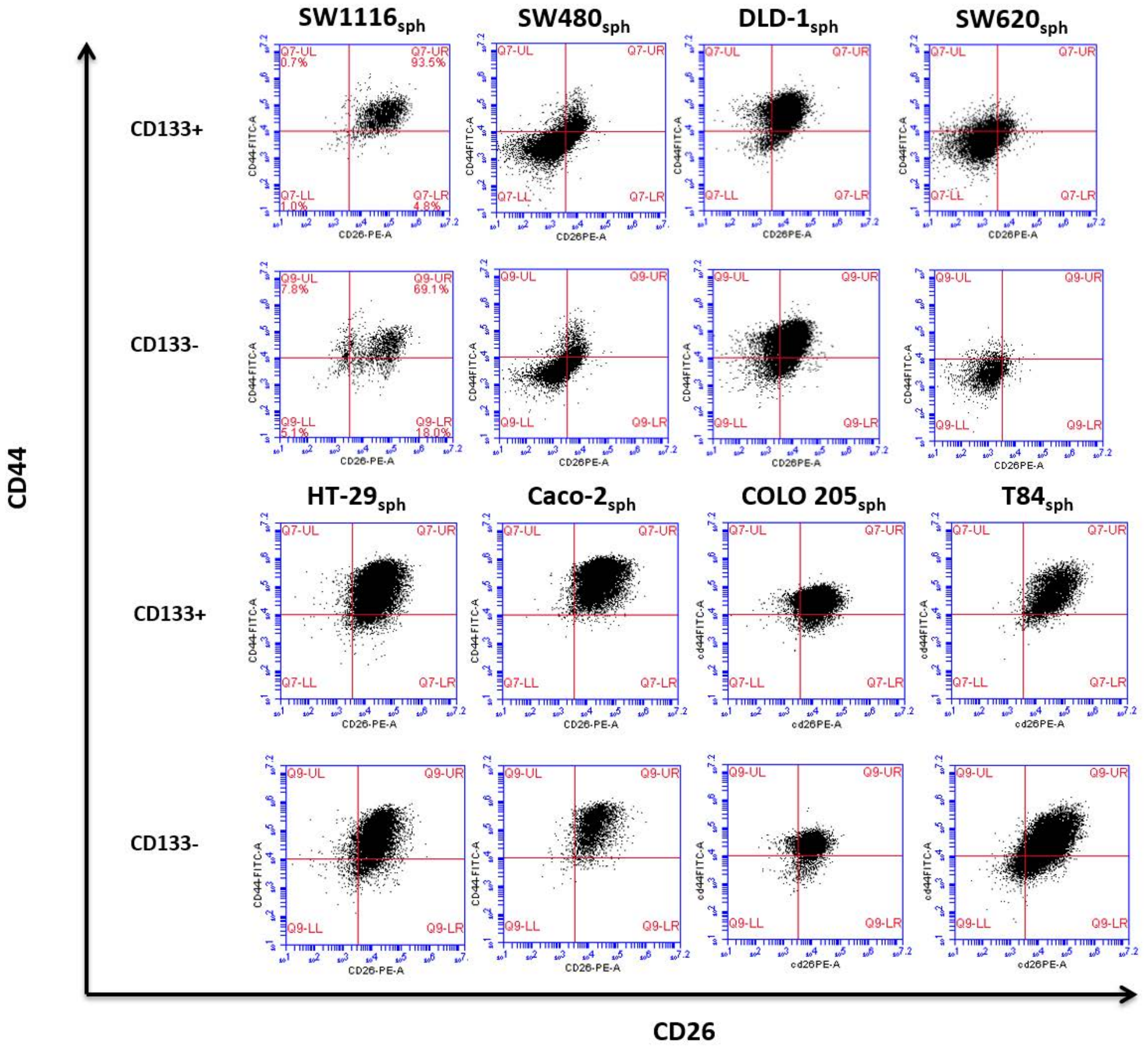


**Fig S4.** Expression of CD44 and CD26 in sphere-derived cells E-cadherin$^{+}$ CD133$^{+}$ or CD133$^{-}$ subsets. Orange bar marks the presence of CD26$^{high}$ population as defined in cell line dot plots.

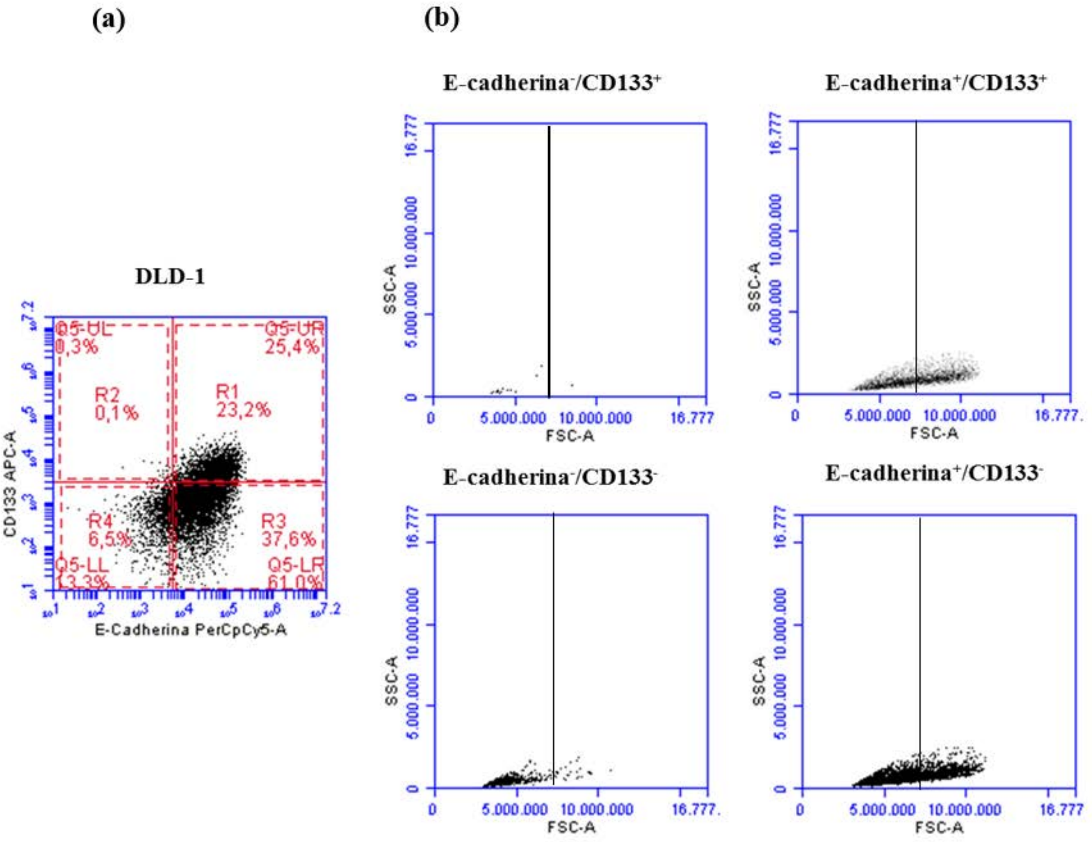


**Fig S5.** Physical (FCS *vs* SSC) dot plot analyses of sphere-derived cells gated for E-cadherin *vs* CD133 expression. a) Dot plot analysis of E-cadherin *vs* CD133 expression in DLD-1$_{sph;}$ .b) Physical gating (FCS *vs* SSC) of the four regions (1 to 4) from the E-cadherin *vs* CD133 dot plot.

Biology Open • Supplementary information

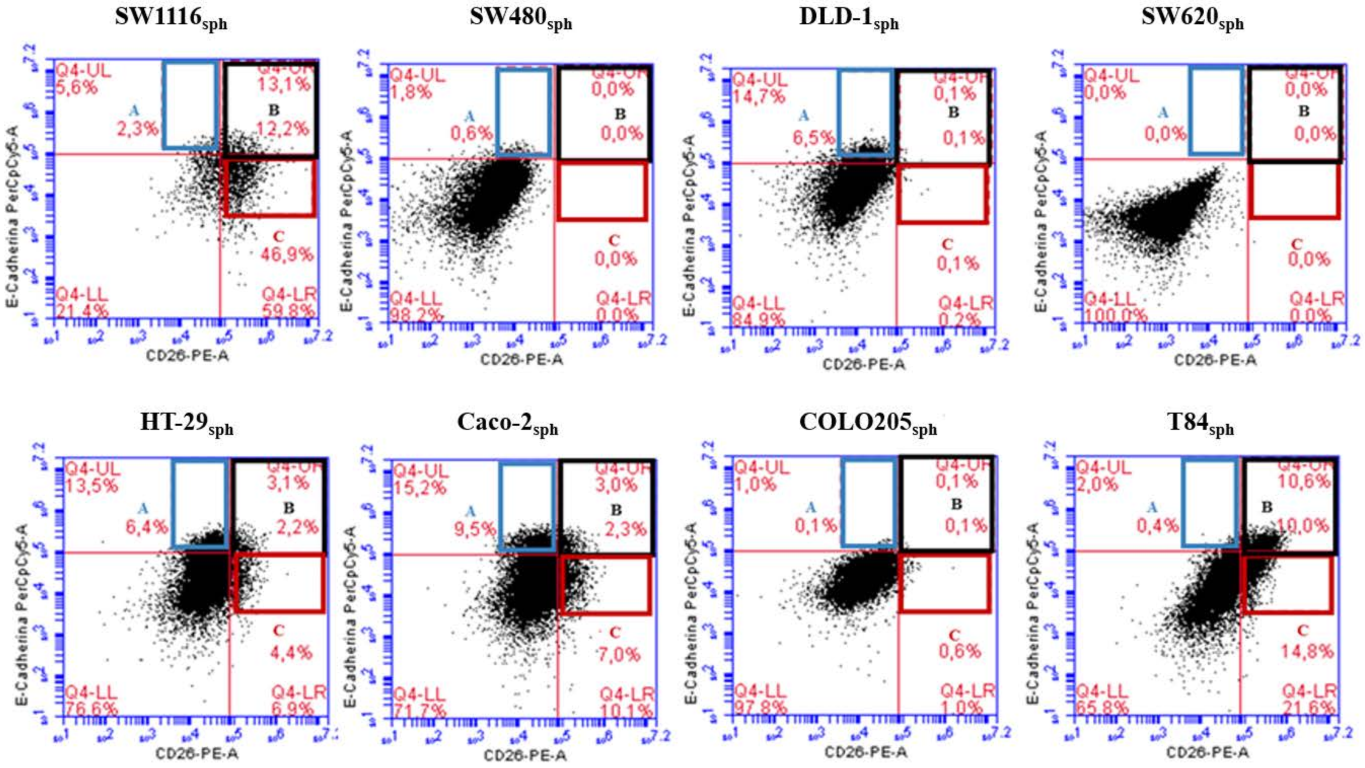


**Fig S6.** Analysis of CD26 and E-cadherin expression in sphere-derived cells. $T84_{sph}$ showed a subset with a stronger coexpression ($CD26^{high}$ $E\text{-cadherin}^{high}$, region B).

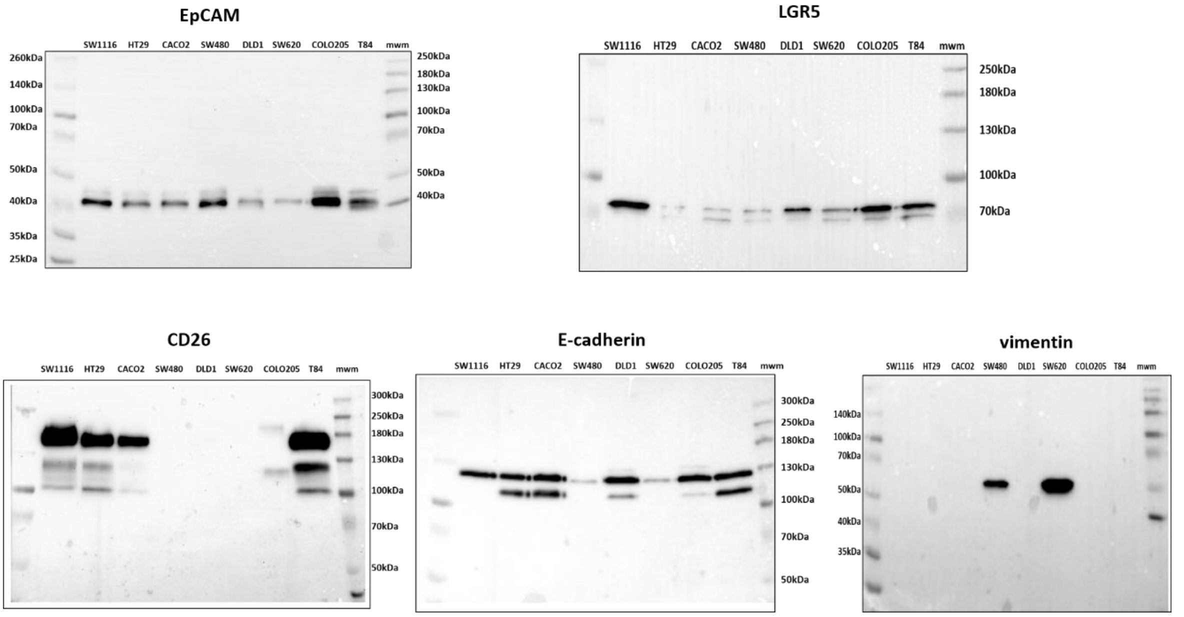


**Fig S7.** Full-length blots of expression of different markers in the eight human colon cancer cell lines analysed (which were shown in different order in Fig 1).

Biology Open • Supplementary information

# Supplemental Tables

## Table S1. Colon cancer cell lines origin

| Cell line | Disease | Dukes Stage | Derived from | References |
|---|---|---|---|---|
| **SW1116** | Colorectal adenocarcinoma | A | Primary tumor | (a) |
| **SW480** | Colorectal adenocarcinoma | B | Primary tumor | (a) |
| **DLD-1** | Colorectal adenocarcinoma | C | Primary tumor | (b) |
| **SW620** | Colorectal adenocarcinoma | C | Metastatic site: Lymph node | (a) |
| **HT-29** | Colorectal adenocarcinoma | n.i. | Primary tumor | (c) |
| **Caco-2** | Colorectal adenocarcinoma | n.i. | Primary tumor | (d) |
| **COLO 205** | Colorectal adenocarcinoma | D | Metastatic site: ascites | (e) |
| **T84** | Colorectal carcinoma | n.i. | Metastatic site: lung | (f) |

n.i.: Not indicated in ATCC.

a. Zimmerer RM, Korn P, Demougin P, Kampmann A, Kokemüller H, Eckardt AM, Gellrich NC, Tavassol F. Functional features of cancer stem cells in melanoma cell lines. Cancer Cell Int. 2013; 6;13(1):78. doi: 10.1186/1475-2867-13-78.

b. Leibovitz A, Stinson JC, McCombs WB 3rd, McCoy CE, Mazur KC, Mabry ND. Classification of human colorectal adenocarcinoma cell lines. Cancer Res. 1976; 36(12):4562-9.

c. Chen TR, Hay RJ, Macy ML. Intercellular karyotypic similarity in near-diploid cell lines of human tumor origins. Cancer Genet. Cytogenet. 1983; 10: 351-362.

d. Fogh J. Human tumor cells in vitro. New York: Plenum Press. 1975.

e. Goodfellow M, Fogh JM, Orfeo T. One hundred and twenty-seven cultured human tumor cell lines producing tumors in nude mice. J. Natl. Cancer Inst. 1977; 59: 221-226.

f. Semple TU, Quinn LA, Woods LK, Moore GE. Tumor and lymphoid cell lines from a patient with carcinoma of the colon for a cytotoxicity model. Cancer Res. 1978; 38: 1345-1355.

# Table S2. Frequencies of autofluorescent cells in colon cancer cell lines.

| **Cell line** | **(-R)** % M ± s.d. | **(+R)** % M ± s.d. |
|---|---|---|
| SW1116 | n.d. | n.d. |
| SW480 | 0.3 ± 0.001 | 1 ± 0.001 |
| DLD-1 | 0.4 ± 0.001 | 0.6 ± 0.001 |
| SW620 | 0.2 ± 0 | 0.2 ± 0 |
| HT-29 | 2.9 ± 0.003 | 3.6 ± 0.006 |
| Caco-2 | 0.7 ± 0.001 | 1 ± 0.001 |
| COLO205 | 0.5 ± 0.001 | 0.7 ± 0.001 |
| T84 | n.d. | n.d. |

(-R) Without riboflavin; (+R) With riboflavin

n.d.: Not determined

M: Mean of three experiments

s.d.: Standard deviation

# Table S3. Frequencies of the subpopulations that express two markers in the colon cancer cell lines.

| | CD133+ | | | CD26+ | | CD44+ | LGR5+ |
|---|---|---|---|---|---|---|---|
| Cell line | CD44+ % M ± s.d. | CD26+ % M ± s.d. | E-cadherin+ % M ± s.d. | CD44+ % M ± s.d. | E-cadherin+ % M ± s.d. | E-cadherin+ % M ± s.d. | EpCAM+ % M ± s.d. |
| SW1116 | 3.9 ± 2.7 | 12.8 ± 8.0 | 11.3 ± 6.7 | 25.0 ± 7.3 | 56.3 ± 7.5 | 16.8 ± 7.2 | 10.2 ± 1 |
| SW480 | 2.9 ± 3.2 | 0.7 ± 0.3 | 2.8 ± 0.7 | 2.1 ± 0.5 | 2.3 ± 0.8 | 33.8 ± 15.9 | 9 ± 0.1 |
| DLD1 | 11.6 ± 8.1 | 9.2 ± 6.8 | 11.3 ± 12.5 | 25.1 ± 7.8 | 17.2 ± 8.0 | 29.3 ± 6.0 | 9 ± 1 |
| SW620 | 1.5 ± 1.6 | 0.9 ± 1.1 | 14.9 ± 7.3 | 0.1 ± 0 | 0.4 ± 0.3 | 2.0 ± 0.9 | 3.7 ± 1.3 |
| HT-29 | 53.3 ± 16.1 | 47.0 ± 14.5 | 34.7 ± 10.6 | 64.5 ± 7.7 | 69.0 ± 3.2 | 62.7 ± 4.3 | 9.1 ± 4.1 |
| Caco-2 | 83.4 ± 1.7 | 78.7 ± 3.2 | 66.4 ± 22.8 | 84.9 ± 4.9 | 83.3 ± 8.9 | 87.7 ± 5.1 | 15.9 ± 6.4 |
| COLO205 | 10.6 ± 9.4 | 32.1 ± 15.3 | 56.5 ± 27.1 | 2.6 ± 1.4 | 68.1 ± 13.9 | 2.5 ± 2.7 | 8.9 ± 3.5 |
| T84 | 1.3 ± 1.4 | 2.0 ± 1.9 | 4.2 ± 4.1 | 30.8 ± 16.5 | 82.1 ± 6.2 | 19.8 ± 6.1 | 25.8 ± 4.4 |

M: Mean of three experiments

s.d.: Standard deviation

Biology Open • Supplementary information

# Table S4. Frequencies of CD26$^{high}$ in colon cancer cell lines.

| **Cell line** | **CD26$^{high}$** % M ± s.d. |
|---|---|
| SW1116 | 41.9 ± 9.2 |
| SW480 | - |
| DLD-1 | 0.1 ± 0.1 |
| SW620 | - |
| HT-29 | 2.1 ± 0.4 |
| Caco-2 | 65.3 ± 4.6 |
| COLO205 | 0.1 ± 0.1 |
| T84 | 12.0 ± 2.9 |

M: Mean of three experiments

s.d.: Standard deviation

-: Not detected

# Table S5. Frequencies of CD26/E-cadherin subsets in colon cancer cell lines.

| Cell line | $CD26^{high}$ | | $E\text{-cadherin}^{high}$ |
|---|---|---|---|
| | $E\text{-cadherin}^{high}$ % M ± s.d. | $E\text{-cadherin}^{low}$ % M ± s.d. | $CD26^{low}$ % M ± s.d. |
| SW1116 | 7.8 ± 5.3 | 15.3 ± 5.7 | 0.8 ± 0.7 |
| SW480 | - | - | - |
| DLD-1 | - | 0.1 ± 0.1 | 6.9 ± 5.4 |
| SW620 | - | - | 0.125 ± 0.25 |
| HT-29 | 1.30 ± 1.07 | 0.45 ± 0.24 | 22.03 ± 16.93 |
| Caco-2 | 6.0 ± 5.3 | 12.7 ± 22.7 | 35.4 ± 23.3 |
| COLO205 | 0.1 ± 0.2 | - | 55.3 ± 22.4 |
| T84 | 2.4 ± 1.6 | 11.2 ± 6.0 | 1.2 ± 0.8 |

M: Mean of three experiments

s.d.: Standard deviation

-: Not detected

# Table S6. Frequencies of CD133/E-cadherin subsets in colon cancer cell lines.

| Cell line | $CD133^{high}$ | | $E\text{-cadherin}^{high}$ |
|---|---|---|---|
| | $E\text{-cadherin}^{high}$ % M ± s.d. | $E\text{-cadherin}^{low}$ % M ± s.d. | $CD133^{low}$ % M ± s.d. |
| SW1116 | 0.1 ± 0.2 | 0.2 ± 0.2 | 1.8 ± 1.9 |
| SW480 | - | - | 0.05 ± 0.06 |
| DLD-1 | - | - | 4.85 ± 4.24 |
| SW620 | - | 0.50 ± 0.63 | 0.30 ± 0.54 |
| HT-29 | 0.03 ± 0.05 | 0.25 ± 0.38 | 10.43 ± 7.40 |
| Caco-2 | 1.95 ± 2.47 | 4.30 ± 5.19 | 42.88 ± 18.89 |
| COLO205 | 0.10 ± 0.14 | 0.08 ± 0.10 | 1.0 ± 0.82 |
| T84 | - | - | 0.18 ± 0.05 |

M: Mean of three experiments

s.d.: Standard deviation

-: Not detected

**Table S7. Properties of the spheres obtained from the colon cancer cell lines.**

| Cell line | Sphere formation | Number of generations | Cells/mL | Radius of spheres |
|---|---|---|---|---|
| SW1116 | Yes | 1 | 1st Generation: $2x10^4$ | 64.46 µm |
| SW480 | Yes | 3 | 1st Generation: $9,5x10^4$<br>2nd Generation: $6x10^4$<br>3rd Generation: $12x10^4$ | 56.27 µm |
| DLD-1 | Yes | 3 | 1st Generation: $11x10^4$<br>2nd Generation: $14x10^4$<br>3rd Generation: $12x10^4$ | 94.13 µm |
| SW620 | Yes | 3 | 1st Generation: $9x10^4$<br>2nd Generation: $14x10^4$<br>3rd Generation: $4x10^4$ | 85.6 µm |
| HT-29 | Yes | 3 | 1st Generation: $12x10^4$<br>2nd Generation: $12x10^4$<br>3rd Generation: $12x10^4$ | 139.74 µm |
| Caco-2 | Yes | 3 | 1st Generation: $10x10^4$<br>2nd Generation: $8x10^4$<br>3rd Generation: $8x10^4$ | 85.6 µm |
| COLO205 | Yes | 3 | 1st Generation: $5x10^4$<br>2nd Generation: $5x10^4$ | 54.63 µm |

| | | | | |
|---|---|---|---|---|
| | | | 3rd Generation: $5x10^4$ | |
| T84 | Yes | 3 | 1st Generation: $3x10^4$<br>2nd Generation: $5x10^4$<br>3rd Generation: $10x10^4$ | 91.40 µm |

Biology Open • Supplementary information

**Table S8. Frequencies of LGR5$^+$/EpCAM$^{low}$ and EpCAM$^{high}$ subsets in cells disaggregated from spheres grown from colon cancer cell lines.**

| **Cell line$_{sph}$** | **EpCAM$^{high}$/LGR5$^+$** % M ± s.d. | **EpCAM$^{low}$/LGR5$^+$** % M ± s.d. |
|---|---|---|
| SW1116 | 56 ± 10.8 | 3.3 ± 2.6 |
| SW480 | 3.2 ± 1.7 | 6.5 ± 4.9 |
| DLD-1 | 8.23 ± 9.68 | 6.10 ± 9.28 |
| SW620 | 0.70 ± 0.98 | 1.46 ± 1.51 |
| HT-29 | 15.60 ± 13.73 | 13.34 ± 15.41 |
| Caco-2 | 6.85 ± 8.85 | 5.33 ± 6.06 |
| COLO205 | 10.75 ± 7.84 | 12.08 ± 14.80 |
| T84 | 13.84 ± 15.77 | 7.06 ± 6.09 |

Cell line$_{sph}$: spheres derived-cells for each cell line

M: Mean of three experiments

s.d.: Standard deviation

Biology Open • Supplementary information

**Table S9. Frequencies of CD26$^{high}$ in cells disaggregated from spheres grown from colon cancer cell lines.**

| **Cell line$_{sph}$** | **CD26$^{high}$** % M ± s.d. |
|---|---|
| SW1116 | 45.7 ± 1.5 |
| SW480 | - |
| DLD-1 | 0.2 ± 0.2 |
| SW620 | - |
| HT-29 | 8.4 ± 0.2 |
| Caco-2 | 11.5 ± 0.4 |
| COLO205 | 0.9 ± 0.1 |
| T84 | 30.1 ± 0.1 |

Cell line$_{sph}$: spheres derived-cells for each cell line

M: Mean of three experiments

s.d.: Standard deviation

-: Not detected

Biology Open • Supplementary information

**Table S10. Frequencies of CD26/CD133 subsets in cells disaggregated from spheres grown from colon cancer cell lines.**

| Cell line$_{sph}$ | CD26$^{high}$ | | CD133$^{high}$ |
|---|---|---|---|
| | CD133$^{high}$ % M ± s.d. | CD133$^{low}$ % M ± s.d. | CD26$^{low}$ % M ± s.d. |
| SW1116 | 0.15 ± 0.06 | 13.70 ± 5.53 | 0.28 ± 0.13 |
| SW480 | - | - | 1.03 ± 1.92 |
| DLD-1 | - | 0.05 ± 0.06 | 0.05 ± 0.10 |
| SW620 | - | - | 1.05 ± 2.10 |
| HT-29 | 0.30 ± 0.35 | 3.45 ± 1.69 | 0.43 ± 0.39 |
| Caco-2 | 0.65 ± 0.87 | 4.00 ± 3.41 | 0.58 ± 0.57 |
| COLO205 | 0,10 ± 0.14 | 0.2 ± 0 | 0.45 ± 0.07 |
| T84 | 0.03 ± 0.05 | 2.00 ± 2.71 | 0.05 ± 0.06 |

Cell line$_{sph}$: spheres derived-cells for each cell line

M: Mean of three experiments

s.d.: Standard deviation

-: Not detected

Biology Open • Supplementary information

**Table S11. Frequencies of CD26/E-cadherin subsets in cells disaggregated from spheres grown from colon cancer cell lines.**

| Cell $line_{sph}$ | $CD26^{high}$ | | $E\text{-}cadherin^{high}$ |
|---|---|---|---|
| | $E\text{-}cadherin^{high}$ % M ± s.d. | $E\text{-}cadherin^{low}$ % M ± s.d. | $CD26^{low}$ % M ± s.d. |
| SW1116 | 3.17 ± 6.01 | 39.67 ± 7.92 | 0.57 ± 1.15 |
| SW480 | 0.025 ± 0.05 | 0.025 ± 0.05 | 0.15 ± 0.3 |
| DLD-1 | 0.05 ± 0.06 | 0.05 ± 0.06 | 2.37 ± 2.95 |
| SW620 | - | - | - |
| HT-29 | 0.75 ± 0.98 | 7.68 ± 7.52 | 2.78 ± 3.28 |
| Caco-2 | 0.65 ± 1.10 | 4.05 ± 2.29 | 2.37 ± 4.75 |
| COLO205 | 0.025 ± 0.05 | 0.2 ± 0.28 | 0.45 ± 0.83 |
| T84 | 2.55 ± 4.97 | 11.25 ± 8.50 | 0.43 ± 0.61 |

Cell $line_{sph}$: spheres derived-cells for each cell line

M: Mean of three experiments

s.d.: Standard deviation

-: Not detected

Biology Open • Supplementary information
